# Regulatory Requirements for the Development of Second-Entry Semisolid Topical Products in the European Union

**DOI:** 10.3390/pharmaceutics15020601

**Published:** 2023-02-10

**Authors:** Alfredo García-Arieta, John Gordon, Luther Gwaza, Virginia Merino, Víctor Mangas-Sanjuan

**Affiliations:** 1 Área de Farmacocinética y Medicamentos Genéricos, División de Farmacología y Evaluación Clínica, Departamento de Medicamentos de Uso Humano, Agencia Española de Medicamentos y Productos Sanitarios, 28022 Madrid, Spain; 2 Division of Biopharmaceutics Evaluation, Bureau of Pharmaceutical Sciences, Pharmaceutical Drugs Directorate, Health Canada, Ottawa, ON K1A 0K9, Canada; 3 Norms and Standards for Pharmaceuticals, Health Products Policy and Standards, Access to Medicines and Health Products Division, World Health Organization, 1211 Geneva, Switzerland; 4 Department of Pharmacy and Pharmaceutical Technology and Parasitology, University of Valencia, 46100 Valencia, Spain; 5 Interuniversity Research Institute for Molecular Recognition and Technological Development, Polytechnic University of Valencia—University of Valencia, 46022 Valencia, Spain

**Keywords:** topical, cutaneous, stepwise approach, qualitative sameness, quantitative similarity, microstructure, semisolid, simple formulation, complex formulation, biowaiver

## Abstract

The development of second-entry topical products is hampered by several factors. The excipient composition should be similar to the reference product because excipients may also contribute to efficacy. Conventional pharmacokinetic bioequivalence studies were not considered acceptable because drug concentrations are measured downstream after the site of action. There was no agreed methodology to characterize the microstructure of semisolids, and waivers of therapeutic equivalence studies with clinical endpoints were not possible. Only the vasoconstrictor assay for corticosteroids was accepted as a surrogate. This paper describes the implementation of the European Union’s stepwise approach for locally acting products to cutaneous products, discusses the equivalence requirements of the EMA Draft Guideline on the Quality and Equivalence of Topical Products, and compares them with the US Food and Drug Administration recommendations. Step 1 includes the possibility of waivers for simple formulations based on in vitro data only (Q1 + Q2 + Q3 + IVRT). Step 2 includes step 1 requirements plus a kinetic study (TS/IVPT/PKBE) to compare the local availability of complex formulations. Step 3 refers to clinical studies with pharmacodynamic/clinical endpoints. As excipients may affect the local tolerability and efficacy of the products, the similarity of excipient composition is required in all steps, except where clinical endpoints are compared.

## 1. Introduction

The development and regulatory approval of second-entry (generic/hybrid) topical products, like many other locally acting products, is hampered by two main factors. First, the approach employed for systemically acting drugs based on the demonstration of equivalent systemic exposure [1], i.e., plasma concentration–time profiles using, e.g., area under the curve and the maximum concentration as primary pharmacokinetic (PK) parameters, was considered unsuitable for locally acting products; this is because the site of action is upstream from the systemic circulation. In the European Union (EU), in contrast to the United States (US), this obstacle has been removed because the similarity in the systemic exposure can now be considered as a surrogate for the similarity of drug levels at the site of action, even if the site of action is upstream, as long as the absorption occurs at the site of action and it is not saturated [2,3]. However, although this has facilitated the development of locally acting products in the gastrointestinal tract or orally inhaled products, this approach is rarely applicable for topical products, e.g., etofenamate or diclofenac, since plasma levels are frequently undetectable or, at least, a complete plasma concentration–time profile is not obtained. Second, since many topical products are semisolid, even when the qualitative and quantitative composition of the test and the reference products are identical, the manufacturing process might provoke differences in the microstructure of the semisolid, which might affect the in vivo drug release and mean that it cannot be characterized sufficiently. Consequently, it was not possible to ensure the similarity and subsequent therapeutic equivalence through in vitro methods. In conclusion, clinical trials were considered essential to demonstrate therapeutic equivalence [4].

It has been agreed recently that if the microstructure of the semisolid is sufficiently different to affect the in vivo release of the active substance from the semisolid formulation, the physicochemical properties of the semisolid formulations and their in vitro release should differ [5,6]. Therefore, if the physicochemical properties of the semisolid are similar, some semisolid products, e.g., some single-phase gels or ointments where the drug is in solution, could be considered therapeutically equivalent. For other more complex semisolids, e.g., emulsions, the in vitro approach needs to be complemented with in vivo or ex vivo data addressing the local bioavailability of the drug in human skin. Consequently, the stepwise approach employed for systemically acting and other locally acting products in the EU, i.e., step 1: biowaiver based on in vitro data, step 2: PK data to reflect local availability at the site of action upstream or downstream and step 3: pharmacodynamic (PD) or clinical endpoints, is presently also possible for topical products in the EU [5].

This work aims to summarize the regulatory requirements for the developing generic/hybrids of topical products in the EU and compare them with the US Food and Drug Administration (FDA) regulatory framework. In this respect, the first difference is that for the US, FDA topical products, other than solutions, are classified as complex products; meanwhile, the term complex is used in the EMA draft guideline to identify formulations that require a comparison not only with in vitro data, but also with kinetic studies to address the local availability of the active substance.

## 2. Legal Basis

The legal basis for second-entry products in the EU differs from the legal basis for these products in the US. In the US, they are considered as generics, whereas in the EU, the generic medicinal products, defined in article 10.1 of Directive 2001/83/CE [7], are presently limited to systemically acting products that demonstrate equivalence through bioavailability studies.

For second-entry locally acting products, the legal basis in the EU should be the “hybrid application” according to article 10.3 of Directive 2001/83/CE [7]. When the EU Directive was elaborated, therapeutic equivalence studies with clinical or PD endpoints were essential to demonstrate therapeutic equivalence. In the cases where a waiver of these studies is possible, e.g., a topical solution with the same qualitative and quantitative composition of excipients, the legal basis should also be the “hybrid application” to keep the same legal basis for all locally applied and locally acting products, irrespective of whether the waiver is applicable or not.

In this paper, we refer to generic/hybrid products that contain the same active substance with the same strength, dosage form, route of administration and instructions for use/labelling. The hybrid legal basis is also applicable to many other products. All those that demonstrate similarity to the reference medicinal products but do not fulfil the conditions of generic products, e.g., different strength, different dosage form, except for oral immediate-release dosage forms whose strength is expressed in the same way, different routes of administration, etc., are also submitted as hybrid applications.

Taking into account that the locally acting products are presently assessed with a stepwise approach similar to the systemically acting drugs, the legal basis of the Directive 2011/83/CE could be revised. Generic products could include not only systemically acting products, but also locally acting products that demonstrate therapeutic equivalence through (a) in vitro data (step 1) due to their high degree of similarity, (b) systemic and/or local bioavailability studies (step 2) that ensure sufficiently similar levels in the site of action, and (c) those that demonstrate therapeutic equivalence through clinical trials with PD, e.g., vasoconstriction assay for corticosteroids, or clinical endpoints as long as these studies are considered to be sufficiently discriminative. Further, studies without the necessary assay sensitivity could be considered as a method to demonstrate the efficacy and safety of the second-entry product; however, as these are not enough to ensure therapeutic equivalence, these products should be stand-alone applications according to article 8.3—mixed application of a known active substance [7].

## 3. Composition Sameness (Q1/Q2) for the Development of Second-Entry Topical Products

As explained above, the development of second-entry topical products should follow a stepwise approach. Ideally, for ethical and economic reasons, equivalence should be demonstrated with in vitro data in the first step. The main limiting factor that complicates not only the first step (in vitro data for a biowaiver), but also the second step (kinetic permeation as a surrogate of local availability) is the clinical relevance of excipients. In the oral systemically acting products, it is generally assumed that excipients do not affect the bioavailability, efficacy and safety of the medicinal product; this is not always correct [8,9]. For locally acting products, the excipients are known to be relevant, especially in topical products, where, e.g., the occlusive function of some excipients, moisturizers and emollients is known to contribute to the therapeutic effect [10,11] or enhance absorption when evaporating; this increases the drug concentration and its thermodynamic activity by disrupting the skin barrier. Consequently, second-entry products that intend to demonstrate therapeutic equivalence to the reference medicinal product with in vitro data only in step 1, or with kinetic studies in step 2, i.e., without conducting therapeutic equivalence clinical trials, need to employ the same active substance in the same form, e.g., salt, which is not required for generics in the EU, and the same excipients, including grade, in the same or very similar amounts.

There are exceptions to this qualitative (Q1) and quantitative (Q2) sameness of excipient. First, some excipients may differ qualitatively. In the EMA draft guideline [5], excipients whose primary function is not related to product performance, i.e., antioxidants, antimicrobial preservatives, and colors, and do not have any other functions or effect that influences the active substance solubility, thermodynamic activity or bioavailability, and product performance, can differ qualitatively. This draft also accepts different paraffin homologues whose function relates to the vehicle or emolliency, and who do not influence the active substance solubility, thermodynamic activity or bioavailability and product performance. However, these differences might be detected through the physicochemical properties of the semisolid, which would preclude the waiver. Second, small quantitative differences are permitted. The difference in the nominal quantitative composition of the excipients should not be greater than ±5%. For excipients whose function only relates to the vehicle properties or emolliency, differences of 10% are acceptable. For excipients whose function is not related to product performance, i.e., antioxidants, antimicrobial preservatives, and colors, 10% differences are also acceptable, but this limit is not logical since qualitative differences are acceptable. In addition, the obligation to use the same salt form is also questionable if equivalence is demonstrated with kinetic studies.

Furthermore, Q1 and Q2 sameness of excipients, as described above, is also required in case of kinetic similarity (step 2). It has been claimed that these kinetic studies are single-dose studies and differences in absorption after repeated administration might occur if the excipients are different, since the excipients that are absorbed in subsequent applications may dissolve the crystalized drug located in the epidermis and promote the absorption of that drug partly absorbed in a previous application [12,13]. However, it is questionable that these different excipients will promote the absorption of subsequent applications differently without promoting the absorption in the first application differently. Therefore, Q1 and Q2 sameness is necessary because of the direct contribution of excipients to the efficacy, e.g., occlusive or hydrating effect. Consequently, if excipients had no contribution to efficacy, e.g., because the efficacy depends on drug concentrations in the synovial liquid and the product is not indicated for a skin disease or it is known that the vehicle has no contribution to efficacy, the Q1 and Q2 sameness could be waived in step 2, where kinetic data is used to support therapeutic equivalence.

For the US FDA, quantitatively the same implies ≥95%, but ≤105% of the reference concentration or amount [14]. Any qualitative or quantitative deviations from the reference should be accompanied by an appropriate in vivo bioequivalence (BE) study(ies). However, changes are allowed for some excipients, i.e., color agents, preservatives, buffers, and antioxidants, which are exceptionally considered inactive ingredients. Applicants should identify and characterize the differences, and submit information demonstrating that the differences do not affect the efficacy and safety of the product.

## 4. Physicochemical and Structural (Q3) Characterization of Second-Entry Topical Products

### 4.1. General Requirements

The nomenclature for describing the dosage form of topical products, e.g., solutions, suspensions, lotions, gels, creams, ointments, sprays, shampoos, pastes, etc., does not correspond to their structural and physicochemical properties. Therefore, the classification based on the dosage form does not ensure products with similar properties. As indicated by the US FDA: “*a product designated as a cream may be comprised of a classic oil-in-water emulsion microstructure, or it may be an aqueous dispersion of different components. An ointment may be comprised of different types of components with different types of Q3 attributes; as examples, an ointment may have an oleaginous hydrocarbon base as a single phase with particles of suspended active ingredient(s), or it may be a water-in-oil emulsion, or it may be comprised of a polyethylene glycol base. In addition, although lotions are typically considered to be more fluid than creams, this may not always be true, and some creams may contain a substantially greater percent composition of water and volatiles than some lotions. Also, although creams and lotions are typically considered to be emulsions, structural features like globules or droplets may not always be evident, and conversely, some gels may be emulsion dosage forms*” [6].

The US FDA has detailed the following physicochemical properties to characterize topical products [6]:

(a) Appearance and texture, including the look, feel and smell of the dispensed product;
  The EMA draft guideline [5] has indicated only appearance but the scope is the same.
(b) Phase states, including high-resolution micrographs to show the absence or presence of undissolved particles to identify single-phase and multiple-phase products;
(c) Structural organization of the matter, including particle size distribution and crystal habit, and/or emulsion globule size distribution and identification of the type of emulsion, e.g., oil in water or water in oil;
(d) Polymorphic form of the active substance within the product for products with a suspended active substance.
  The EMA draft guideline [5] indicates the particle size distribution and polymorphism for suspensions and the globule size distribution for emulsions, but crystal habit is not mentioned; however, it is mentioned as a factor that may affect bioavailability and should be included in stability studies;
(e) Rheological behavior of liquid and semisolid dosage forms, including
  1. Complete flow curves plotting shear stress vs. shear rate, and viscosity vs. shear rate until low or high-shear plateaus are achieved. Apparent viscosity should be reported at least at low, middle, and high shear rates.
    The EMA draft guideline specifies that these shear stress flow sweep experiments should comprise multiple data points across the range of increasing and decreasing shear rates so that any linear portions of the up-curves or down-curves are clearly identified. In contrast to the US FDA, for the EMA, the resulting curves should be characterized by fitting to (modified) power law equations so that numerical data can be produced [15]; however, the minimum of the three share rates to estimate apparent viscosities is not defined, only the need to measure the apparent viscosity at specified shear rates across the rheograms, e.g., η100, and the quantification of the thixotropic relative area (S_R_) if appropriate.
  2. Yield stress, if the products exhibit plastic flow behavior.
  3. Linear viscoelastic response (storage and loss moduli vs. frequency).
    The EMA draft guideline specifies that viscoelastic storage and loss moduli (G’ and G”), and loss tangent (tan δ) should be determined in these oscillatory strain sweep (shear strain oscillatory amplitude sweep) experiments [15] to obtain parameters that can be compared objectively. The EMA draft guideline also mentions the creep test.
(f) Water activity and/or drying rate, relevant for products with volatile excipients including water;
(g) pH and buffering for products with an aqueous component;
(h) Oleaginous components, which should be characterized according to the tests listed in the US Pharmacopeia;
(i) Specific gravity (density);
(j) Metamorphosis of the product when dispensed from the containers.

The EMA draft guideline defines the pH, buffering capacity, viscosity, density, surface tension and osmolality for solutions and suspensions, and the pH density and rheological behavior for semisolids, although this separation is not so straightforward. The characterization of the oleaginous components, metamorphosis, water activity and drying rate are not included.

Both the US FDA and EMA require that batches of different ages or storage periods should be characterized.

### 4.2. Product-Specific Requirements

The in vitro tests required for the characterization and comparison of the physicochemical properties of these semisolid dosage forms depend on the characteristics of the specific reference product; they should be defined case-by-case, since not all of them are applicable. In this regard, a “Quality Attributes Data Comparison Protocol” should be employed in the EU, as indicated in the reflection paper on statistical methodology for the comparative assessment of quality attributes in drug development [16], in order to pre-define those quality attributes or physicochemical properties that define the semisolid product. This task is facilitated by the product-specific guidances (PSGs) for generic drug development, elaborated by the US FDA [17], since the EMA has not developed any product-specific guideline for topical products at present [18]. The Draft Guideline on the Quality and Equivalence of Topical Products defines only the usual physicochemical properties that characterize a semisolid [5], but additional characteristics may be necessary depending on the composition of the formulation, e.g., characterization of the oleaginous components, metamorphosis, water activity and drying rate. For example, it is necessary to characterize the water activity of those formulations where the solvent activity affects the performance of the formulation. Water activity depends on excipient composition and manufacturing variables and, in addition to the viscosity and thermodynamic activity of the drug, controls the drug diffusion rate within the vehicle and affects drug output from the formulation. Vehicles with low water activity alter the hydrodynamics of skin and cause structural changes in the stratum corneum. Small molecule humectants, such as propylene glycol, retain skin hydrodynamics [19].

The following tables and paragraphs illustrate the diversity within each dosage form type.

#### 4.2.1. Solutions

In the EU, the physicochemical properties to be compared for solutions are pH, buffering capacity, viscosity, density (or specific gravity), surface tension and osmolality [5]. In the USA (see Table 1), in some old PSGs, these parameters are not identified, e.g., [20,21,22,23,24,25,26,27,28,29], but the same can be observed in the most recently revised PSG, e.g., [30,31,32,33]. In the Ciclopirox topical solution PSG, the physicochemical properties refer to the polymeric resin (molecular weight distribution, number of butyl groups/g of resin) [34], because the resin defines the properties of the formulation and the nail coat. In the PSG for Efinaconazole topical solution [35] and Tavaborole topical solution [36], their specific properties are as follows: appearance, specific gravity, viscosity, evaporation (drying) rate and surface tension; pH is added to this list for hydrogen peroxide [37]. Therefore, it can be concluded that the physicochemical properties defined in the EMA draft guideline should be adapted to each specific product. For example, the pH and buffering capacity are not applicable for non-aqueous solutions and the drying rate may be necessary for those products containing volatile solvents.

#### 4.2.2. Suspensions

For suspensions, the EMA draft guideline [5] identifies the same physicochemical parameters as noted above for solutions. Obviously, for drug particles in suspension, additional characterization, in terms of active substance particle size distribution and polymorphic form, including photomicrographs, is required. In addition, an in vitro release test (IVRT) should demonstrate a similar release rate and the total amount released at the end of the study, since the concept of extended pharmaceutical equivalence coined in this draft guideline includes equivalent performance. In the US FDA PSG for betamethasone dipropionate and calcipotriene topical suspension [38], the physicochemical and structural characterization details are as follows: (a) visual appearance and texture; (b) phase states and structural organization of the matter by means of (i) microscopic examination and (ii) particle size distribution, crystal habit, and polymorphic form of the drug substance(s) in the drug product; (c) rheological behavior, which includes (i) characterization of shear stress vs. shear rate and viscosity vs. shear rate to obtain numerical viscosity data at three shear rates (low, medium, and high), (ii) a complete flow curve across the range of attainable shear rates, until low or high shear plateaus are identified and (iii) yield stress values that should be reported if the material tested exhibits plastic flow behavior, but the linear viscoelastic response does not have to be reported; (d) specific gravity; and (e) equivalent rate of betamethasone dipropionate and calcipotriene release that must be shown in an IVRT according to the draft guidance in vitro Release Test Studies for Topical Drug Products Submitted in ANDAs [39]. In the PSG for Spinosad topical suspension [40], pH and water activity are added to the previous list, but the analyses of particle size distribution, crystal habit and polymorphic form are not required (see Table 2). The above rheological parameters are required in the EMA draft guideline for semisolids, but not for suspensions. However, as stated above, the nomenclature used to describe the dosage form of topical products, e.g., solutions, suspensions, gels, lotions, creams, ointments, sprays, shampoos, pastes, etc., does not correspond to the compositional, physicochemical, or structural attributes of the drug product. Therefore, some suspensions may need the characterization defined for semisolids in the EMA draft guideline.

In contrast, for ciclopirox topical suspension [41] and Ketoconazole shampoo (suspension) [42], a waiver is not possible in the US FDA. In this regard, the waiver of the EMA draft guideline is always applicable unless

(a) the drug has a narrow therapeutic index (NTI), but none have been classified as NTI in this route of administration;
(b) the drug exhibits dose-related systemic toxicity, but this can be addressed by comparing systemic exposure with conventional PK BE studies;
(c) the means by which the active substance reaches the local site of action is not established or understood; this is not expected presently and, moreover, it might be claimed that if the formulation is considered to be simple and an extended pharmaceutical equivalence is met, the applied product will be therapeutically equivalent in any case;
(d) the method of administration is not the same, which might be a limitation only if the application device/method is patented;
(e) the product cannot be fully characterized with respect to quality attributes, but this is not foreseen if the reference product has been authorized recently;
(f) it is not possible to measure a quantifiable permeation kinetic or PD event for the product; however, a stratum corneum sampling/tape stripping (TS) study might be used; and
(g) in vitro and in vivo permeation kinetic and PD studies are not applicable or are considered insufficiently predictive of clinical response, e.g., products indicated for the treatment of open wounds and ulcers, which would apply only for complex formulations as explained above. If the formulation is considered simple and extended pharmaceutical equivalence is met, the applied product will be therapeutically equivalent in any case. Therefore, the main limitation is the possibility of developing sensitive IVRT or an in vitro permeation test (IVPT), or the reproducibility of the TS technique.

As indicated in the last point, the difficulty of developing second-entry topical products increases when considering products applied to the mucous membrane or damaged skin, where the skin models described below (IVPT, PK BE and TS) may not be representative. In those cases, e.g., in vaginal semisolids, the same principles could be followed, but using skin models as surrogates for mucous membranes may not be possible. Therefore, if the formulation is considered “simple”, the same Q1, Q2 and Q3, including IVRT, may be enough to demonstrate equivalence. On the contrary, if the formulation is complex, the most convenient methodology to demonstrate therapeutic equivalence is to conduct a PK BE study if the drug is absorbed into the systemic circulation from the site of action, if that absorption is not saturated, or at least if its less-than-proportional increase for the dose–AUC relationship is closer to proportionality than the dose–therapeutic response curve. In the cases where the semisolid is applied both in the skin and mucous membranes, the skin model could be used for the cutaneous indications and the extrapolation to the mucous membrane indications or site of application may need to be justified.

#### 4.2.3. Gels

For gels, the physicochemical and structural characterization always includes visual appearance and texture, phase state and structural organization of the matter by microscopic examination, particle size distribution and crystal habit. Considered also are the polymorphic form of the drug substance(s) if it is in suspension in the drug product, or the globule size distribution if it contains an emulsion (see Table 3).

The rheological behavior always includes the characterization of shear stress vs. shear rate and viscosity vs. shear rate, in order to obtain numerical viscosity data at three shear rates (low, medium, and high) and the yield stress if the material tested exhibits plastic flow behavior. However, the complete flow curve across the range of attainable shear rates is not always required and the linear viscoelastic response is only required in a few cases (see Table 3). Specific gravity is always required, but other physicochemical parameters, such as pH and drying rate, are recommended case by case (see Table 3). For some gels, therapeutic equivalence trials are still required, e.g., [64,65], or a PD blanching assay [66,67].

#### 4.2.4. Ointments

For lipophilic ointments, it is necessary to characterize the oleaginous components in a few cases (see Table 4), in addition to the previously described parameters, such as particle size distribution, crystal habit, and polymorphic form when particles are in suspension, and globule size distribution for emulsions in the ointment (see Table 4). Although it is included in Table 4, the biowaiver for the combination of betamethasone dipropionate and calcipotriene is only applicable for the calcipotriene component because a PD vasoconstriction study is recommended for betamethasone dipropionate [68].

For hydrophilic ointments, the characterization is slightly simpler, e.g., mupirocin ointment containing polyethylene glycol 400 and polyethylene glycol 3350 [69].

For some old drug products, grandfathered drugs, gentamicin sulfate [70], nystatin [71], triamcinolone acetonide [72,73] and the combination of the last two [74], the recommendations for these ointments do not include the Q1 and Q2 sameness. Only the physicochemical characterization is needed, but the parameters to compare them are not defined in the PSG. The same can be seen in some creams (Table 5).

**Table 4:** US FDA PSG recommendations for the biowaiver of ointments.

Drug	RS	Q	AT	M	PS	GS	AV	FC	YS	LVR	WA	pH	SG	DR	OC	IVRT	IVPT
Acyclovir [75]	18604	√	√	√	√		√	√	√				√			√	
Betamethasone Dipropionate; Calcipotriene [68]	21852	√	√	√	√		√	√	√				√		√	√	√
Calcipotriene [76]	20273	√	√	√	√	√	√	√	√	√			√		√	√	√
Crisaborole [77]	207695	√	√	√		√	√	√	√	√			√		√	√	√
Gentamicin Sulfate [70]	62351																
Mupirocin [69]	50591	√	√	√			√	√	√				√			√	
Nitroglycerin [78]	21359	√	√	√		√	√	√	√	√			√		√		
Nystatin [71]	62124																
Nystatin; Triamcinolone Acetonide [74]	63305																
Tacrolimus [79]	50777	√	√	√		√	√	√	√	√			√		√	√	√
Tacrolimus [80]	50777	√	√	√		√	√	√	√	√			√		√	√	√
Tirbanibulin [81]	213189	√	√	√			√	√	√				√			√	
Triamcinolone Acetonide [71]	087385																
Triamcinolone Acetonide [73]	08995																

RS: RLD or RS Number. Q: Q1 and Q2. AT: Appearance and texture. M: Micrographs. PS: Particle size distribution, crystal habit and polymorphic form of the active substance. GS: Globule size distribution. AV: Shear stress vs. shear rate, viscosity vs. share rate and apparent viscosity at least at low, middle, and high shear rates. FC: Complete flow curve across the range of attainable shear rates until low or high shear plateaus. YS: Yield stress. LVR: Linear viscoelastic response. WA: Water activity. SG: Specific gravity. DR: Drying rate. OC: Characterization of oleaginous components. √: Recommended.

#### 4.2.5. Creams

All the above properties need to be addressed for creams depending on the reference product characteristics (see Table 5).

For example, in acyclovir cream, it is necessary to characterize the particle size distribution, crystal habit, and polymorphic form of acyclovir in the drug product, but not the globule size distribution [82]. The contrary is required for ammonium lactate [84], and both are needed for others such as the combination of acyclovir and hydrocortisone [83], calcipotriene [88] and docosanol [89]. A complete rheological characterization, pH and specific gravity are generally required for creams. On the contrary, water activity and drying rate should be characterized in a few cases. Although included in Table 5, the biowaiver for the combination of betamethasone dipropionate and calcipotriene is only applicable for the calcipotriene component because a PD vasoconstriction study is recommended for betamethasone dipropionate [85]. The waiver is not possible for a fluorouracil product containing microspheres [111].

#### 4.2.6. Lotions

Similar requirements can be found for lotions as for emulsions, e.g., appearance and texture, complete rheological characterization, specific gravity, and particle size distribution and polymorphism [112], globule size distribution [113,114], or both [115] (Table 6). pH needs to be compared in all cases, except in the combination of miconazole nitrate, white petrolatum, and zinc oxide due to its fatty nature, for which the oleaginous components need to be characterized [116]. The drying rate only needs to be compared for ammonium lactate [112]. These recommendations do not apply to triamcinolone acetonide, which, as a grandfathered drug, only needs a comparison of undefined physicochemical properties without Q1 and Q2 sameness [117]. In addition, some lotions are recommended based only on Q1 and Q2 sameness in solutions [118,119].

#### 4.2.7. Other Topical Dosage Forms

To illustrate the diversity of dosage forms and tests to characterize topical products, it can be highlighted that the tests mentioned above differ from those required for topical aerosol-foam [124], which include the following:

(a) Microscopic birefringence analysis of the dispensed foam after complete collapse, in order to determine whether any crystals of undissolved active substance form during dispensing.
(b) Time to break (from dispensing to complete foam collapse) analysis, conducted at 30 °C, 33 °C, 35 °C, and 40 °C under controlled relative humidity conditions.
(c) Weight per volume of uncollapsed foam.

Nystatin powders [125] are waived based on comparative physicochemical and structural (Q3) characterization, without defining the necessary in vitro tests. Ciclopirox [126] and clobetasol propionate [127] shampoos can be waived based on Q1 and Q2 sameness, like solutions. The same criterion is applied for sprays of clobetasol propionate [128] and desoximetasone [129], as well as clindamycin [130] and erythromycin [131] swabs. For glycopyrronium tosylate cloth [132], the same dimensions and content are recommended, and it is necessary to compare pH and absorbency.

Importantly, the US FDA PSG does not waive all products with the same systematic approach due to the complexity of these products. Therapeutic equivalence trials with clinical endpoints or PD endpoints are required without the option of a biowaiver for some suspensions [41], gels [133] (even where other gels of the same drug can be waived), ointments [134], lotions [120] (even where other lotions of the same drug can be waived), creams [111] (even where other creams of the same drug can be waived), and aerosol-foam [135] (even where the other topical dosage forms such as cream and ointment of the same drug can be waived). In contrast, the EMA draft guideline intends to apply the general principles to all products, and it is the responsibility of the applicant to develop the necessary methods for the waiver. The fact that a well-established PD model, such as skin blanching, or that a clinical trial design and endpoint are available should not be a reason to preclude the biowaiver. Only the lack of adequate methodology for the comparison, e.g., IVRT for all semisolid simple and complex formulations, IVPT, TS, or PK BE studies for complex formulations, should preclude the biowaiver; this is because the means by which the active substance reaches the local site of action should be known, and the approved products should be fully characterized with respect to quality attributes.

## 5. Statistical Comparison of Physicochemical Properties

A comparison of basic Q3 characterizations for both test and reference products is recommended/required to demonstrate that a test product and its reference are not only the same dosage form, but also structurally similar to support that there is no relevant difference in Q3 attributes that may affect the local or the systemic BE.

First, it is essential to highlight that, for the US FDA, the physicochemical properties are compared based on ranges. Each attribute of the test product has to be demonstrated by the applicant to be within the range characterized for that attribute of the reference standard for the topical product, or determined by the Agency to be within the acceptable variability for the reference standard of the topical product [6]. This approach does not seem to control the type I error required for an inferential analysis, and the FDA only recommends using a 90% confidence interval (CI) for the comparison between the test and reference to conclude equivalence in the analysis of the IVRT [39]. In contrast, in the EMA draft guideline [5], an inferential analysis based on 90% CI is required for all comparisons.

The critical quality attributes should be compared according to a pre-planned “Quality Attributes Data Comparison Protocol”, since adequate pre-planning is needed to avoid data-driven analyses and biased post hoc decisions. This protocol should define the objective and the context of the quality attributes data comparison, i.e., the waiver of a clinical therapeutic equivalence trial based on demonstrated in vitro similarity (physicochemical properties and IVRT, when applicable). Demonstration of equivalence in all the quality attributes under comparison is necessary to obtain a waiver.

This protocol should define and justify the critical quality attributes that are to be compared, i.e., the criticality assessment, what criteria is employed to conclude that the products are equivalent in each quality attribute under comparison, i.e., the similarity condition (e.g., the ratio of the geometric means do not differ more than ±10%, which may differ depending on the quality attribute), and the similarity criterion for each quality attribute, i.e., the statistical methods to conduct the comparisons (e.g., the calculation of the 90% confidence interval for the ratio of the geometric means of test and reference product).

Multiple critical quality attributes should be characterized and compared, as described above. Some of them are qualitative, e.g., appearance, texture, polymorphic form, microscopic images, shear stress vs. shear rate curves, viscosity vs. share rate curves, flow curves, storage and loss modulus vs. frequency curves, while others are quantitative, e.g., pH, viscosity, density/specific gravity, surface tension, osmolality, zero shear viscosity, apparent viscosities at different shear rates, yield stress, storage modulus and loss modulus, water activity, and drying rate. These are surrogates for what consumer’s feel regarding the cooling sensation and smoothness, which contribute to sensorial equivalence and are believed to contribute to the therapeutic outcome. For example, apparent yield stress represents the strength that has to be applied to extract the formulation from the container, and zero shear viscosity is related to the consistency in the container at rest, which influences the dosing when the container is pressed. The apparent viscosities at higher shear rates represent the consistency when the semisolid is applied to the skin and its spreadability, which impacts the width of the applied layer. Thixotropic relative area, storage and loss moduli inform on the microstructure of the semisolids. Although showing equivalence in multiple parameters increases the probability of a failure at random, no difference should exist if the composition of Q1 and Q2 is the same. Therefore, the problem of showing equivalence seems to be associated with the variability in the measurements and the representativeness of the limited batches under comparison. A consistent failure to show equivalence would indicate differences caused by the different manufacturing process or excipient supplier.

In principle, these comparisons should be conducted prospectively, based on the information gained during product development. The observed inter- and intra-batch variability, as well as the variability in the testing methods, should be used to define the number of batches of test and reference to be compared, the number of units per batch to be selected, and the number of replicates within each unit to be measured, to reach the desired power to show equivalence according to the expected differences in the quality attributes between test and reference and the pre-defined acceptance range in the similarity condition.

However, if this information is already available before pharmaceutical development, based on the literature or internal data, e.g., via the development of similar products, the investigations conducted during the pharmaceutical development could also be used for the comparability exercise. In this case, the “Quality Attributes Data Comparison Protocol” should be elaborated before the pharmaceutical development starts and the pharmaceutical development should be conducted in accordance with the “Quality Attributes Data Comparison Protocol”, e.g., sampling strategy, number of batches, units per batch and replicates to be analyzed for product characterization.

In any case, the protocol should define a sampling strategy that ensures the representativeness of the samples to be analyzed for a meaningful interpretation of the results in this inferential equivalence analysis, and to avoid the data-driven selection of batches/units. It is acknowledged that these comparisons are limited to non-random samples of batches of the reference product available in the market. Therefore, it is recommended that all the batches available in the market are selected if they are few, or a representative sample with different ages is selected if multiple batches are available in the market. Similarly, for the test product, the representativeness of the selected batches should be discussed and the age of the batches at the time of analysis should be similar to those of the reference product if the age of the batch is a relevant factor explaining the variability in the data. In cases where the quality attributes change significantly during the shelf-life, it might be convenient to demonstrate similarity at the beginning and the end of the shelf-life.

Although the EMA draft guideline [5] recommends for quantitative characteristics the analysis of untransformed data based on differences if the data follows the Normal distribution, the log-transformation of the data may be better for expressing the results as a percentage, since ±10% of the comparator product mean is defined as the acceptance range. Using the ratio instead of the difference would be consistent with the analysis conducted for the IVRT, IVPT and TS. Furthermore, due to the large variability observed in some of these quality attributes [136], widening the acceptance range based on the total variability in the reference product would be convenient to avoid unnecessary large sample sizes [137].

## 6. In vitro Release Tests (IVRT)

In addition to showing Q1 sameness and Q2 and Q3 similarity, it is necessary to demonstrate similar in vitro release. For suspensions, this is usually conducted with an in vitro dissolution test and for semisolids with a vertical diffusion cell (Franz cell), but other equipment can be used, e.g., immersion cell. The IVRT should demonstrate, in a pilot study, that the release rate is drug concentration-dependent and is discriminative to altered formulations with changes in critical quality attributes, e.g., particle size distribution or rheological properties, critical manufacturing variables or quantitative excipient composition; however, the complete omission of one excipient is not accepted.

The IVRT should demonstrate similarity in the cumulative amount released and the release rate with the 90% CI for the ratio of means of the test and the reference products within an acceptance range of 90.00–111.11%, based on a parametric approach according to the EMA draft guideline, and there should be a lag time difference within ±10% [5]. Taking into account that these limits are narrow, the minimum sample of 12 samples may not be enough, and a proper sample size calculation is necessary. Twelve samples of test and reference provide 80% power to conclude similarity within ±10% if the inter-sample CV is 12.5% and there is no difference between the test and reference products. If the variability is larger, or if the release from the test and reference is not identical, 12 samples will not have enough (i.e., 80%) power to show equivalence under the assumption of log-normal distribution.

In contrast, the US FDA recommends a non-parametric approach with a ±25% (75.00–133.33%) acceptance range for the release rate only in the Guidance for Industry for Scale-up and Postapproval Changes of Nonsterile Semisolid Dosage Forms [138]. This analysis is conducted first with six samples per product and if equivalence is not shown, the sample size is increased to twelve additional samples. The type I error does not seem to be adjusted in this two-stage design.

The US FDA has published a detailed draft guidance on how to develop, validate and conduct the IVRT [39], whereas this topic is addressed in Annex I of the EMA draft guideline [5]. In this annex, it is required that the duration of IVRT should be sufficient to characterize the release profile, where ideally, at least 70% of the active substance applied is released; however, 30% is sufficient, which has been omitted. Steady-state release kinetics can typically be assumed under conditions when the dose depletion is less than 30%, although, for some topical products, steady-state release kinetics may continue to be observed at higher percentage dose depletions [39]. Although the US FDA draft guidance is more detailed and recommends linearity, with r^2^ ≥ 0.97 for the estimation of the release rate as the slope of the amount released vs. the square root of time, and the proportionality of changes in the release rate as a function of drug concentration in the formulation with r^2^ ≥ 0.95, the EMA draft guideline only requires r^2^ > 0.90 in the latter. In contrast, the EMA draft indicates that this proportionality slope should be statistically different from zero. The EMA draft requires that the method is discriminative to changes in the Q2 excipient composition, manufacturing processes, particle size or rheology. In contrast, the US FDA only requires this supplemental selectivity when feasible. The EMA requires accuracy in the application methodology of the product into the donor compartment with differences within ±5% between samples, but this is not defined in the US FDA draft. The reproducibility between operators should be <10% in the EMA draft, but reproducibility should be ≤15% in the US FDA draft. The US FDA recommends 4–6-h tests, and the EMA requires six samples at least hourly; however, the duration is not defined. The US FDA recommends formulations with 50% and 150% of content to assess the proportionality of the release rate versus the product concentration, but the EMA draft guideline does not define concentration levels. The US FDA indicates that the analytical method to measure drug concentration in the receptor solution should be validated according to requirements for biological samples, not in line with QC methods, and highlights the importance of separate protocols and reports for the bioanalytical method and IVRT. Finally, the US FDA indicates that this documentation should be submitted in Module 5 of the CTD (ICH Common Technical Document) [139], like comparative bioavailability and BE studies.

## 7. Kinetic Studies to Address Local Availability

These studies represent step 2 of the stepwise approach of the EMA. This step is required when the formulation is considered complex, e.g., emulsions, those containing excipients that enhance drug penetration or complex excipients where different suppliers or grades may affect the in vivo performance of the active substance. In contrast to the stepwise approach for other types of products, e.g., orally inhaled, where the fulfillment of step 1 is not required if step 2 requirements are met, in topical products, fulfillment of step 1 is also required, and no exception is defined in the EMA draft guideline [5]. This is because excipients in, e.g., orally inhaled products, are less diverse and have only a carrier function; however, in topical products, excipients may also contribute to the efficacy of the product.

While the US FDA only accepts in vitro permeation tests to assess the local availability (rate and extent of absorption, not the distribution, metabolism and excretion that occurs in vivo) of the active substance in the skin [140], the EMA has considered within the draft guideline three types of studies: stratum corneum sampling or tape stripping, in vitro permeation tests and conventional PK BE studies.

Stratum corneum sampling based on TS was investigated by the US FDA and discarded because it was considered unreliable [141], but it is accepted in Japan [142]. The results were unreliable in the US FDA studies because different areas for sampling were used in the two laboratories conducting the comparison, and one of the products was distributed laterally. In addition, this technique is also criticized because removing the first two tapes to exclude the unabsorbed drug may exclude the absorbed drug. Alternatively, the inclusion of these initial tapes and the improvement in the cleaning method of the surface of the skin before starting sampling is questionable since the product may remain in the skin furrows. Furthermore, the absorption may also occur through the hair follicle and the sebaceous glands, which is not taken into account by the stratum corneum sampling technique. However, this technique may play a role if the laboratory can standardize the methodology and if it is used only for drugs that remain in the stratum corneum and do not diffuse through the viable epidermis into the dermis, which would make the IVPT unfeasible. It may be applicable also for drugs that diffuse deeper, but for those cases, IVPT seems to be more feasible.

Furthermore, the methodology has been simplified with respect to the dermal PK studies proposed initially by the US FDA, where a complete profile of concentrations versus time was obtained. Presently, the methodology only intends to compare the concentrations at two sampling times, one for uptake and another for clearance, with replicate determinations to increase precision and reduce the sample size [143,144,145,146,147]. It is acknowledged that only a few centers with experience can conduct these studies. Many factors complicate the generalized implementation of this methodology, e.g., the type of glue tape, the weighting and grouping of the tapes, which confer a huge variability to these studies. More importantly, it is essential to include a negative control to give internal validity to these studies.

IVPT are currently accepted both by US FDA and EMA drafts and has become the preferred method. The US FDA has issued a detailed draft guidance on IVPT [140], where method development, method validation with a pilot study and the comparison with the pivotal IVPT are clearly distinguished; this also includes the distinct concepts of SOP, protocol and reports, and the difference between the validation of the IVPT method and the validation of the bioanalytical method used to measure the drug in the receptor solution. The negative control is used during method validation to demonstrate selectivity, but the example of the EMA draft guideline based on a formulation with 50% of the proposed strengths is described in the US FDA draft as the least adequate strategy to address sensitivity. Modulating the dose amount or the dose duration is preferred to produce the desired effect of altering the permeation profiles. Therefore, it would be preferable to alter the excipient composition or the manufacturing process for selectivity. The EMA draft guideline defines a maximum duration of 24 h, unless membrane integrity is demonstrated for longer periods. In contrast, the US FDA draft does not define any limit and describes the possibility of 48 h. More importantly, the dose duration may be shorter than the study duration to obtain a complete flux profile where a decline follows the maximum flux in subsequent time points, which is not mentioned in the EMA draft guideline. The EMA guideline defines a limit for the accuracy of the methodology for product application to the donor compartment (±5%) and requires accounting for the mass balance with a ±10% limit; it also suggests the quantification of the drug in the different skin layers, which the US FDA does not mention. The EMA draft guideline also requires measuring the membrane integrity after the experiments, but this is not recommended by the US FDA draft. Unless justified, the receptor solution should be an aqueous buffer for the EMA draft, but it should contain 0.1% Oleth-20 to enhance the solubility of hydrophobic drugs, according to the US FDA draft. The number of donors should be at least 12 in the EMA draft guideline, and there is no limit in the US FDA for the pivotal study (4–6 donors in the pilot study). The number of replicates per donor should be 2 per treatment for the EMA draft and 4 (with a minimum of 3 for the inclusion of the donor in the statistical analysis) for the US FDA draft. The statistical analysis is similar to conventional BE studies, with an acceptance range of 80–125% for the primary PK parameters (maximum rate of absorption and total amount permeated at the end of the experiment); however, in case of high variability, the scaling or widening is conducted differently. In addition, the EMA draft requires the lag-times to not differ by more than 10% and the reporting of the time of maximum flux.

IVPT is especially relevant when the site of action is located within the viable dermis of the skin. When the site for action is located in the dermis or beyond, the systemic exposure may also be suitable for comparing test and reference products. Since absorption into the systemic circulation occurs from the dermis, it can be assumed that if systemic exposure is equivalent, it is because the exposure in the dermis is also equivalent, even if systemic exposure is downstream; this is unless the absorption in the dermis is saturated, which is not considered the limiting factor for absorption, since it is usually the stratum corneum. For active substances acting in deeper tissues, e.g., synovial liquid in the knee, the diffusion from the dermis into those deeper tissues and the systemic circulation may occur by two different routes, but both reflect the concentrations at the site of action. The clinical response reflects the concentration at the site of action with low sensitivity and the systemic exposure reflects with more sensitivity, although indirectly, the concentrations at the dermis and consequently at the site of action; this is enough for a relative comparison between test and reference.

Conventional PK BE studies are not suitable to compare the local availability in the skin or beyond, according to the US FDA criterion, because the systemic circulation is downstream. Therefore, it is used only to assess the systemic safety of the medicinal products that produce measurable systemic concentrations. Then, they are recommended in addition to the IVPT for doxepin hydrochloride cream and ivermectin cream [90,95]. In contrast, in the EMA draft guideline, they are alternatives. Although it would be nice to possess all of the kinetic information, it is not essential. If one of the methods fails and the other succeeds in showing similarity, a sound justification would be necessary. The only case where the US FDA recommends only a PK BE study is the lidocaine/prilocaine cream [148]. In this case, the test products do not need to be Q1, Q2 and Q3. The obligation to use the same salt for the active substance and to demonstrate Q1, Q2 and Q3 similarity can be questioned when BE is shown with a PK BE study. It can be claimed that, if any difference in excipient may alter the solubilization of the crystalized active substance after repeated administration, that solubilization differences might also be expected after the single dose. Using the same excipients in similar amounts is convenient to ensure a similar local safety profile, but Q1 differences and larger Q2 differences could be accepted if equivalence is shown with PK BE studies and excipients do not contribute to efficacy. Q3 differences could be acceptable if they do not affect parameters such as patient perception, residence time and occlusivity. As these perceptions and parameters are so difficult to judge, it is convenient to use as many Q1, Q2 and Q3 similar test products as possible.

Other methodologies, e.g., dermal microdialysis, are not covered in the EMA guideline, but they could be used if adequately validated. The same can be applied to pharmacodynamic models, where only the vasoconstriction assay for corticosteroids and the in vivo microbial decolonization studies for antiseptics are mentioned.

## 8. Pharmacodynamic and Clinical Endpoints

Although trials with PD and clinical endpoints are out of the scope of this paper, it is important to address the Q1, Q2 and Q3 requirements when these endpoints are used to demonstrate therapeutic equivalence.

In the US FDA PSGs, the in vivo option, based on one study with a clinical endpoint, does not imply the need to have Q1, Q2 and/or Q3 similar formulations, e.g., azelaic acid topical cream [149]. The same applies for the EU, since the third step can be applied when the previous steps have failed, and the local and systemic safety profile would be addressed as secondary endpoints in the efficacy study, demonstrating therapeutic equivalence.

In the case of the PD vasoconstriction assay, the US FDA PSG does not mention the need to have Q1, Q2 and/or Q3 similar formulations, e.g., [150], in line with existing guidance [151] and draft guidance [152]. However, in the EU, the Question and Answer (Q&A) in the guideline Clinical Investigation of Corticosteroids Intended for Use on the Skin [153] states that the vasoconstriction or blanching assay is only an acceptable surrogate for the therapeutic equivalence trials with a clinical endpoint if the generic medicinal product possesses the same or a similar qualitative and quantitative composition to that of the reference product. This is justified because, in dermatology, the vehicle itself may influence the disorder in aspects not covered by the vasoconstriction, as the potency of the corticosteroid alone cannot predict the efficacy of the entire preparation. Differences in excipients have to be considered case by case. In the case of only minor changes, e.g., slight differences in the quantity of the same excipients in generic applications, the vasoconstriction assay can be accepted instead of clinical efficacy studies. However, qualitative changes in the composition imply the need for clinical efficacy data. In addition, this Q&A document indicates that local tolerance studies are usually required even when the compositions are Q1 and Q2 similar, and that the preferred design is a double-blind vehicle-controlled repeated application study in healthy volunteers. Fortunately, this requirement has never been applied, even in formulations with some qualitative differences, which indicates that it needs to be revised.

## 9. Decision Tree for the Development of Second-Entry Topical Products

Once the different types of studies to demonstrate equivalence for topically applied and acting products have been described, this section proposes a decision tree to apply the principles mentioned above.

**Figure 1:**
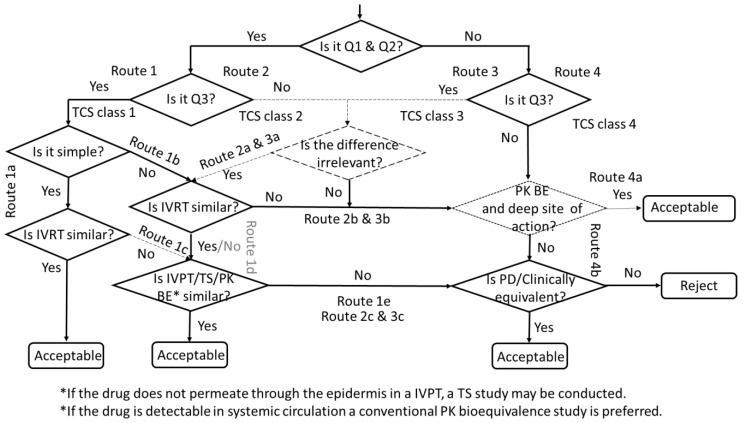
Decision tree for the development of second-entry topical products.

The development of these topical products should be approached in steps. In the first step, shown on the left-hand side of Figure 1, the Q1 and Q2 composition of excipients should be copied as much as possible to be considered sufficiently similar within the range of difference described above. Then, Q3 similarity should be demonstrated to ensure that the microstructure of the product is similar to that of the reference product (Route 1). If the product is considered a simple formulation with the criterion defined in the EMA draft guideline [5], the demonstration of a similar in vitro release (IVRT) is enough to obtain a waiver to obtain a marketing authorization (Route 1a/Step 1).

This is similar to the Topical Classification System (TCS) Class 1, defined by Shah et al. [154], which refers to drug products, whereas the Biopharmaceutics Classification System refers to drugs. Moreover, these authors summarize the whole microstructure in the IVRT and do not refer to the need to compare the physicochemical properties to ensure a similar microstructure that affects product performance in other ways than the drug release, e.g., residence time in the application site, adhesion, occlusion, ease of application, etc. This TCS considers that the IVRT is enough to ensure therapeutic equivalence, in line with the SUPAC-SS recommendations [138]. However, as a different manufacturer is involved with slight differences in the manufacturing process and there is some room for Q1 and Q2 differences within the similarity definition, i.e., ±5%, more evidence than that which the IVRT offers is necessary to conclude Q3 similarity.

If the formulation is not considered simple, e.g., emulsions or formulations containing penetration enhancers, in addition to the IVRT, it is necessary to go into the second step of the European stepwise approach (Route 1b) and to demonstrate similarity in the local availability through one of the kinetic studies (IVPT, TS or PK BE). If the drug does not permeate through the epidermis in an IVPT, a TS study may be conducted, and, if the drug is detectable in the systemic circulation, a conventional PK bioequivalence study is preferred because its methodology is well established; in addition, it could allow larger differences in Q1 and Q2 if the site of action is beyond the skin, or if the excipients have no contribution to the clinical response (see route 4).

These two routes (1a and 1b) are similar to the approaches described in the US FDA PSG, but not identical, because the US FDA does not divide the topical formulations into simple and complex formulations. The US FDA considers topical products other than solutions as complex products, and the recommendations are defined in the PSG. For some creams, e.g., acyclovir [82], an IVPT is recommended in addition to Q1, Q2, Q3 and IVRT, but for others it is not, e.g., ammonium lactate [84]. See Table 2, Table 3, Table 4, Table 5 and Table 6 for products where IVRT or IVRT plus IVPT are recommended. The same can be observed for lotions such as abametapir [113] and benzyl alcohol [114], where the IVPT is not required; however, the globule size distribution of the emulsion should be characterized. On the contrary, it is required for the lotion of halobetasol propionate 0.01% [120].

In the PSG for benzyl alcohol lotion [114] and ivermectin lotion [121], a pediculicide hair tuft assay is required. In the PSG of doxepin HCl cream [90] and ivermectin cream [95], an additional PK study is necessary to address systemic safety. Similarly, the EMA draft guideline requires a pharmacodynamic blanching study for products containing corticosteroids. If IVRT and IVPT are considered enough to ensure that the drug concentrations are equivalent at the site of action in other products, the fact that systemic concentrations are measurable or a validated pharmacodynamic study is feasible should not be a reason to increase the regulatory burden. If Q1, Q2, Q3 and the IVRT are similar for ivermectin lotion, there is no reason to expect a different pediculicide effect. Similarly, for the ivermectin cream, if, in addition to Q1, Q2, Q3 and IVRT, the IVPT is also similar, there is no reason to expect a different systemic absorption. Therefore, the PK study is an alternative to the IVPT according to the EMA draft guideline, not a supplement.

On the left-hand side, a discontinuous line, representing a new proposal, can be considered when the products that comply with Q1, Q2 and Q3 similarity, and are simple formulations, fail to show similar IVRT (Route 1c). This is unlikely if a wide acceptance range is defined, e.g., ±25% by the US FDA SUPAC-SS; however, this may occur if a narrow acceptance range is defined, e.g., ±10% in EMA draft guidance, even if the widening of the acceptance range based on the variability in the reference product is permitted. It might be the case that dermal absorption is regulated by the stratum corneum as the limiting factor and, e.g., the IVPT might conclude similarity. In this scenario, this decision tree proposal is that the IVPT would overrule the IVRT failure.

Similarly, for a complex formulation, if we consider the European stepwise approach, when in vitro data fails, the next step is to investigate the local bioavailability with one of the kinetic studies (Route 1d when IVRT is not similar, grey color). Therefore, it could be argued that even if the IVRT fails, the product might be approvable if the kinetic study can show equivalence.

If the kinetic studies fail, it is necessary to show equivalence with PD or clinical endpoints (Step 3 of the European stepwise approach, route 1e).

The center of the decision tree addresses the scenario where the formulations are Q1 and Q2, but Q3 is not demonstrated (Route 2a), e.g., when there is a failure to demonstrate similarity in any of the physicochemical parameters, but when large differences are not observed. This may represent a US FDA scenario because Q3 is not compared statistically based on 90% CI, but is only based on the ranges of the observations, for which the US FDA has not defined any statistical methodology. In principle, this scenario with Q3 failure requires therapeutic equivalence trials with PD or clinical endpoints. For example, the Q3 differences may alter not only the drug release and absorption, but also the amount applied from the container and the method of application. Only if it were possible to conclude that these differences do not affect the clinical use and its response could the regulatory approval be based on the IVRT, plus one of the kinetic studies. This route is plotted as a discontinuous line as it is only a proposal without regulatory value. This judgement is difficult to make presently due to our limited knowledge and regulatory experience. Both IVRT and IVPT studies would be needed even if the formulation is simple, because additional evidence is needed to compensate for the Q3 failure. Of course, if Q1 and Q2 similarity is fulfilled, but Q3 is notably different and relevant, the waiver is not possible, as Shah et al. [154] proposed for TCS class 2 products (Route 2b). If the IVRT or the IVPT fails, PD or clinical equivalence studies would be necessary (Route 2b or 2c).

The right-hand side of the decision tree describes the scenario where the formulations are Q1 and Q2 dissimilar. In principle, this precludes any biowaiver. If, despite these Q1 and/or Q2 differences, the microstructure was similar (Q3) and it could be demonstrated that the excipient differences do not impact the efficacy and safety of the product (Route 3a), e.g., because there is no penetration enhancer in the formulation and because excipients are considered inert with equivalent function, the similarity in IVRT and one of the kinetic studies could support a waiver. Again, both the IVRT and one of the kinetic studies are necessary, even if the formulation is simple, since additional evidence is needed to compensate for the Q1 and/or Q2 differences. This approach would be similar to the TCS class 3 proposed by Shah et al. [154], but it must be noticed that Q3 is equal to IVRT for these authors. On the contrary, the EMA draft guideline and this decision tree distinguish between physicochemical properties and IVRT. If it were not possible to demonstrate that the excipients do not contribute to the clinical efficacy and safety, route 3b should be followed. If the IVRT or the IVPT of route 3a fails, PD or clinical equivalence studies would be necessary (Route 3b or 3c).

In cases where Q1, Q2 and Q3 are dissimilar (Route 4 at the right-hand side of the decision tree), a clinical study with PD or clinical endpoint is necessary to demonstrate therapeutic equivalence. This represents the TCS class 4 proposed by Shah et al. [154]. However, it must be highlighted that in the EMA Q&A for corticosteroids, similar Q1 and Q2 are required to accept the pharmacodynamic vasoconstriction endpoint as a surrogate, which might be questionable if the drug activity is much higher than any excipient contribution; this is not mentioned by the US FDA. Therefore, a clinical endpoint is needed if the route comes from the right-hand side (Route 3 or 4: Q1 and Q2 dissimilar). If the route comes from the left-hand side (Routes 1 or 2), i.e., Q1 and Q2 similarity with Q3 (and IVRT or IVPT) differences, the vasoconstriction assay can be used.

An exception to this rule might be proposed (plotted with a discontinuous line) if equivalence is shown through a conventional PK study and it is assumed that the systemic circulation reflects the concentrations in the dermis, i.e., if the absorption from the dermis is not saturated, or the site of action is local but beyond the dermis. This is consistent with the US FDA PSG recommendations for lidocaine/prilocaine cream [148].

## 10. Development of Additional Strengths

While the US FDA has developed product-specific guidances for each strength, e.g., tacrolimus 0.1% [79] and 0.03% [80], which implies that the whole development has to be repeated for each strength, the EMA draft guideline includes the possibility of reducing the requirements for the development of the additional strengths.

If several strengths are developed, it may be sufficient to establish equivalence with the most sensitive strengths to detect potential differences between the test and reference. The different strengths should be manufactured by the same manufacturing process and with the same Q1 composition. If the Q1 and Q2 compositions of the different strengths of the test products are similar to the corresponding strengths of the reference product, the waiver of the additional strength can be based on the extended pharmaceutical equivalence between the corresponding strengths of the test and reference, i.e., Q3 and IVRT. This is irrelevant for products where the waiver is based only on the IVRT, such as adapalene 0.1% and 0.3% gels [43,44], but it is relevant for those cases where kinetic studies, e.g., IVPT [79,80] or the PD/clinical endpoint trials, are requested [64].

## 11. Virtual Bioequivalence

Mechanistic models of cutaneous absorption that combine in vitro information regarding the drug substance and the drug product with physiologically based pharmacokinetics (PBPK), allow the in silico investigation of the in vivo effect of formulation differences on local and systemic exposure in what are named “virtual bioequivalence” studies, which can be conducted in healthy volunteers and patient populations because these models can take into account intra-subject and inter-subject variability in populations [155,156]. Cutaneous PBPK models include inter- and intra-subject variability by adding variability into skin physiology parameters, such as skin thickness, pH and blood flow, and virtual subjects of different sex, race, and age groups. Simulation can reflect the cutaneous kinetics of the active moiety after drug application at different anatomic sites, including arms, legs, head, abdomen, and back within the same virtual subject [157].

These mechanistic models can save economic resources and time, but require a large amount of data to develop, optimize and validate. This effort will be compensated only if large, long, expensive and/or unfeasible clinical trials are waived. In addition, when these models are shown to be sufficiently predictive, it can be claimed that the factors that affect active moiety local and systemic bioavailability are known; thus, confidence in the product performance is gained by investigating “what if” scenarios.

In the EU, the use of virtual BE studies does not seem to be necessary, since these virtual studies are intended to substitute for therapeutic equivalence studies with clinical endpoints, which are very infrequent in the EU. These studies are not necessary for simple formulations if the qualitative and quantitative composition is sufficiently similar, as explained above, and if equivalence is shown with an IVRT. For complex formulations, the clinical studies can be waived if equivalence is shown, in addition to the requirements for simple formulations, in an in vitro permeation test, a PK BE study for drugs that are measurable in systemic circulation, or a TS dermato-PK study for drugs that remain in the stratum corneum and do not permeate through the dermis in an IVPT.

If the conditions for a waiver are not met and virtual studies are employed, the predictive value of the PBPK model through the dermal route should be the same as the one required for the oral route. To consider that the in vitro release or permeation method is biopredictive, the predicted local and systemic exposure using those methods should be comparable (±10%) to the observed in vivo PK data [158]. Formulations with different release rates should be tested to demonstrate this predictive ability within the investigated design space. Virtual BE studies are not considered in the EMA draft guideline, but deviations from the guideline are acceptable if justified.

For example, the US FDA has approved a diclofenac gel based on virtual BE [159], but in Spain, a diclofenac gel was approved based on a PK BE study based on systemic exposure [160]. It is assumed that if the levels are equivalent in the systemic circulation, they are also equivalent in the dermal tissue from where the drug is absorbed into the systemic circulation; this is if it is shown that the absorption is not saturated from the site of application and absorption. Once they are equivalent in the dermis, it is assumed that they will also distribute to the deeper sites of action in an equivalent manner.

## 12. Conclusions

The principles defined in the EMA draft guideline on the quality and equivalence of topical products reflects the current state-of-the-art methods and are similar to those of the US-FDA. These principles have been applied in recent years and some products have been approved, which demonstrates that it is applicable; however, it could be convenient to widen the acceptance range according to the observed variability in the reference product. This guideline, when finished and adopted, will consolidate the stepwise approach for locally acting products in the skin. This stepwise approach is slightly different from those used for other locally acting products because the similar Q1 and Q2 excipient composition seems necessary not only in the first (in vitro) step, but also in the second (kinetic) step; it might even be required in the case of PD studies. The third step should be avoided as much as possible because the clinical endpoints tend to be insensitive to large differences in the administered dose, and the large sample sizes required have ethical and practical limitations. When the sensitivity of the clinical or PD endpoints is inadequate, these products should not be considered switchable, i.e., generic/hybrid, but only as prescribable (Art. 8.3 full mixed application).

## Figures and Tables

**Table 1:** US FDA PSG recommendations for the biowaiver of solutions.

Drug	RS	Q1Q2	A	V	pH	SG	DR	ST
Calcipotriene [20]	020611	√						
Clindamycin Phosphate [30]	050537	√						
Clobetasol Propionate [21]	019966	√						
Clotrimazole [31]	018181	√						
Diclofenac sodium [32]	204623	√						
Diclofenac sodium [22]	020947	√						
Efinaconazole [35]	203567	√	√	√		√	√	√
Erythromycin [23]	064187	√						
Fluorouracil [24]	016831	√						
Fluorouracil [25]	016831	√						
Hydrocortisone [26]	081271	√						
Hydrogen peroxide [37]	209305	√		√	√	√	√	√
Minoxidil [27]	019501	√						
Minoxidil [28]	020834	√						
Podofilox [29]	019795	√						
Tavaborole [36]	204427	√		√		√	√	√
Tretinoin [33]	016921	√	√					

RS: RLD or RS Number. A: Appearance. V: Viscosity. SG: Specific gravity. DR: Drying rate. ST: Surface tension. √: Recommended.

**Table 2:** US FDA PSG recommendations for the biowaiver of suspensions.

Drug	RS	Q	AT	M	PS	GS	AV	FC	YS	LVR	WA	pH	SG	DR	OC	IVRT	IVPT
Betamethasone Dipropionate; Calcipotriene [38]	22185	√	√	√	√		√	√	√				√			√	
Spinosad [40]	22408	√	√	√			√	√	√		√	√	√			√	

RS: RLD or RS Number. Q: Q1 and Q2. AT: Appearance and texture. M: Micrographs. PS: Particle size distribution, crystal habit and polymorphic form of the active substance. GS: Globule size distribution. AV: Shear stress vs. shear rate, viscosity vs. share rate and apparent viscosity at least at low, middle, and high shear rates. FC: Complete flow curve across the range of attainable shear rates until low or high shear plateaus. YS: Yield stress. LVR: Linear viscoelastic response. WA: Water activity. SG: Specific gravity. DR: Drying rate. OC: Characterization of oleaginous components. √: Recommended.

**Table 3:** US FDA PSG recommendations for the biowaiver of gels.

Drug	RS	Q	AT	M	PS	GP	AV	FC	YS	LVR	WA	pH	SG	DR	OC	IVRT	IVPT
Adapalene [43]	20380	√	√	√	√		√	√	√			√	√			√	
Adapalene [44]	21753	√	√	√	√		√	√	√			√	√			√	
Adapalene, Benzoyl Peroxide [45]	22320	√	√	√	√		√	√	√			√	√			√	
Adapalene, Benzoyl Peroxide [46]	207917	√	√	√	√		√	√	√			√	√			√	
Bexarotene [47]	21056	√	√	√			√		√				√	√		√	
Clindamycin Phosphate [48]	50782	√	√	√			√	√	√			√	√			√	
Clindamycin Phosphate [49]	50615	√	√	√			√	√	√			√	√			√	
Clindamycin Phosphate; Tretinoin [50]	50802	√	√	√	√		√	√	√			√	√			√	
Clindamycin Phosphate; Tretinoin [51]	50803	√	√	√			√	√	√			√	√			√	
Dapsone [52]	207154	√	√	√	√		√	√	√			√	√			√	√
Dapsone [53]	21794	√	√	√	√		√	√	√			√	√			√	√
Diclofenac Sodium [54]	22122	√	√	√		√	√	√	√	√		√	√			√	√
Diclofenac Sodium [55]	21005	√	√	√			√	√	√			√	√			√	
Ketoconazole [56]	21946	√	√	√			√	√	√				√			√	
Metronidazole [57]	19737	√	√	√			√	√	√			√	√			√	
Metronidazole [58]	21789	√	√	√			√	√	√			√	√			√	
Podofilox [59]	20529	√	√	√			√		√			√	√	√		√	
Tazarotene [60]	20600	√	√	√	√		√	√	√			√	√			√	
Tretinoin [61]	17579	√	√	√			√		√				√	√		√	
Tretinoin [62]	22070	√	√	√	√		√	√	√			√	√			√	
Tretinoin [63]	17955	√	√	√			√		√				√	√		√	

RS: RLD or RS Number. Q: Q1 and Q2. AT: Appearance and texture. M: Micrographs. PS: Particle size distribution, crystal habit and polymorphic form of the active substance. GS: Globule size distribution. AV: Shear stress vs. shear rate, viscosity vs. share rate and apparent viscosity at least at low, middle, and high shear rates. FC: Complete flow curve across the range of attainable shear rates until low or high shear plateaus. YS: Yield strass. LVR: Linear viscoelastic response. WA: Water activity. SG: Specific gravity. DR: Drying rate. OC: Characterization of oleaginous components. √: Recommended.

**Table 5:** US FDA PSG recommendations for the biowaiver of creams.

**Drug**	**RS**	**Q**	**AT**	**M**	**PS**	**GS**	**AV**	**FC**	**YS**	**LVR**	**WA**	**pH**	**SG**	**DR**	**OC**	**IVRT**	**IVPT**
Acyclovir [82]	21478	√	√	√	√		√	√	√	√	√	√	√			√	√
Acyclovir, Hydrocortisone [83]	22436	√	√	√	√	√	√	√	√	√	√	√	√			√	√
Ammonium Lactate [84]	20508	√	√	√		√	√	√	√	√		√	√	√		√	
Betamethasone Dipropionate; Calcipotriene [85]	213422	√	√	√		√	√	√	√	√	√	√	√			√	√
Butenafine Hydrochloride [86]	20524	√	√	√		√	√	√	√	√		√	√	√		√	
Butenafine Hydrochloride [87]	21307	√	√	√		√	√	√	√	√		√	√	√		√	
Calcipotriene [88]	20554	√	√	√	√	√	√	√	√	√		√	√			√	√
Docosanol [89]	20941	√	√	√	√	√	√	√	√	√		√	√	√		√	
Doxepin Hydrochloride [90]	20126	√	√	√		√	√	√	√	√		√	√			√	√
Fluocinolone Acetonide [91]	12787																
Fluorouracil [92]	16988	√	√	√		√	√	√	√	√		√	√			√	√
Fluorouracil [93]	22259	√	√	√		√	√	√	√	√		√	√			√	√
Gentamicin Sulfate [94]	62307																
Ivermectin [95]	206255	√	√	√		√	√	√	√	√		√	√			√	√
Ketoconazole [96]	19084	√	√	√	√	√	√	√	√	√		√	√	√		√	
Luliconazole [97]	204153	√	√	√	√	√	√	√	√	√		√	√	√		√	
Metronidazole [98]	20531	√	√	√		√	√	√	√	√		√	√			√	√
Metronidazole [99]	20743	√	√	√	√	√	√	√	√	√		√	√			√	√
Mupirocin Calcium [100]	50746	√	√	√	√	√	√	√	√	√	√	√	√	√		√	
Nystatin [101]	64022																
Nystatin; Triamcinolone Acetonide [102]	62364																
Oxymetazoline Hydrochloride [103]	208552	√	√	√		√	√	√	√	√		√	√			√	√
Ozenoxacin [104]	208945	√	√	√	√	√	√	√	√	√		√	√			√	√
Penciclovir [105]	20629	√	√	√	√	√	√	√	√	√	√	√	√			√	√
Pimecrolimus [106]	21302	√	√	√	√	√	√	√	√	√		√	√			√	√
Silver Sulfadiazine [107]	17381	√	√	√	√	√	√	√	√	√		√	√	√		√	
Tazarotene [108]	21184	√	√	√		√	√	√	√	√		√	√			√	√
Tazarotene [109]	21184	√	√	√		√	√	√	√	√		√	√			√	√
Triamcinolone Acetonide [110]	11601																

RS: RLD or RS Number. Q: Q1 and Q2. AT: Appearance and texture. M: Micrographs. PS: Particle size distribution, crystal habit and polymorphic form of the active substance. GS: Globule size distribution. AV: Shear stress vs. shear rate, viscosity vs. share rate and apparent viscosity at least at low, middle, and high shear rates. FC: Complete flow curve across the range of attainable shear rates until low or high shear plateaus. YS: Yield stress. LVR: Linear viscoelastic response. WA: Water activity. SG: Specific gravity. DR: Drying rate. OC: Characterization of oleaginous components. √: Recommended.

**Table 6:** US FDA PSG recommendations for the biowaiver of lotions.

Drug	RS	Q	AT	M	PS	GS	AV	FC	YS	LVR	WA	pH	SG	DR	OC	IVRT	IVPT
Abametapir [113]	206966	√	√	√		√	√	√	√	√		√	√			√	
Ammonium Lactate [112]	19155	√	√	√	√		√	√	√	√		√	√	√		√	
Benzyl Alcohol [114]	22129	√	√	√		√	√	√	√	√		√	√			√	
Clindamycin Phosphate [115]	50600	√	√	√	√	√	√	√	√	√	√	√	√			√	√
Halobetasol Propionate [120]	209355	√	√	√		√	√	√	√	√		√	√			√	√
Ivermectin [121]	202736	√	√	√		√	√	√	√	√		√	√			√	
Malathion [118]	018613	√															
Metronidazole [122]	20901	√	√	√		√	√	√	√	√		√	√			√	√
Miconazole Nitrate; White Petrolatum; Zinc Oxide [116]	21026	√	√	√	√		√	√	√	√			√		√	√	
Mometasone Furoate [119]	019796	√															
Tazarotene [123]	211882	√	√	√		√	√	√	√	√		√	√			√	√
Triamcinolone Acetonide [117]	11602																

RS: RLD or RS Number. Q: Q1 and Q2. AT: Appearance and texture. M: Micrographs. PS: Particle size distribution, crystal habit and polymorphic form of the active substance. GS: Globule size distribution. AV: Shear stress vs. shear rate, viscosity vs. share rate and apparent viscosity at least at low, middle, and high shear rates. FC: Complete flow curve across the range of attainable shear rates until low or high shear plateaus. YS: Yield stress. LVR: Linear viscoelastic response. WA: Water activity. SG: Specific gravity. DR: Drying rate. OC: Characterization of oleaginous components. √: Recommended.

## Data Availability

Not applicable.
